# Performance of amikacin inhale: impact of supplemental oxygen and device orientation

**DOI:** 10.1186/cc14200

**Published:** 2015-03-16

**Authors:** N Kadrichu, K Corkery, T Dang, P Challoner

**Affiliations:** 1 Novartis Pharmaceuticals, San Carlos, CA, USA; 2 Nektar Therapeutics, San Francisco, CA, USA

## Introduction

Amikacin Inhale is an integrated drug-device combination in development by Bayer HealthCare through a collaboration with Nektar Therapeutics, to improve clinical outcome in intubated and mechanically ventilated patients with Gram-negative pneumonia. It is available in two configurations: on-vent for intubated patients and hand-held for extubated patients to complete aerosolized antibiotic therapy. Amikacin Inhale is a smart system that consists of the pulmonary drug delivery system with a vibrating mesh nebulizer and the specially formulated Amikacin Inhalation Solution (400 mg every 12 hours for 10 days). The objectives of this study were to evaluate the performance of the Amikacin Inhale hand-held configuration with supplemental O_2_ concentration supplied at different flow rates and in different orientations. We hypothesize that the delivered dose of amikacin will not significantly change with increased O_2_ flow rate or varying orientation.

## Methods

In the hand-held configuration of Amikacin Inhale, amikacin is aerosolized into a holding chamber. Amikacin aerosol is inhaled with ambient air entering the bottom of the chamber through the inhalation valve. Supplemental O_2_ may be supplied through the O_2 _port and mixes with ambient air entering through the inhalation valve. O_2_ concentration and delivered dose at the mouthpiece exit were characterized *in vitro *at various O_2_ flow rates (2 to 10 l/minute). O_2 _concentrations were measured every minute until the end of dosing. A ventilator was connected to the set up to simulate patient breathing. The delivered dose was measured at the exit of the mouthpiece. Drug distribution within the test setup compartments was analyzed using HPLC. The *in vitro *delivered amikacin dose was also measured at nebulizer orientations of 0° and 45° (*n *= 3 per orientation) using a simulated breathing profile with no supplemental O_2_.

## Results

The mean O_2_ concentration ranged from 36 to 70% over 2 to 10 l/minute and was ≥40% at ≥3 l/minute. The delivered dose did not change substantially with increasing enriched O_2_ flow rate (72 to 82% of nominal dose). At 0° and 45° orientations, the delivered dose of amikacin was 74 to 80% and 73 to 76% of the nominal dose (400 mg), respectively.

## Conclusion

Amikacin Inhale was shown *in vitro *to be suitable for extubated patients who require supplemental O_2_. The delivered dose was independent of supplemental O_2_ and device orientation.

